# Association of the inflammatory microenvironment of obesity with pathological complete response in patients with breast cancer receiving neoadjuvant chemotherapy

**DOI:** 10.3389/fendo.2025.1659454

**Published:** 2025-12-05

**Authors:** Dongxu Yang, Keru Ma, Hao Wang, Yang Liu, Yuwei Du, Fang Fang, Xuquan Qin, Dalin Li

**Affiliations:** 1 Department of Breast Surgery, Harbin Medical University Cancer Hospital, Harbin, Heilongjiang, China; 2 Department of General External Medicine, North Hospital, Qiqihar First Hospital, Qiqihar, Heilongjiang, China

**Keywords:** BC, obesity, BMI, PCR, inflammation

## Abstract

**Background:**

The impact of the inflammatory microenvironment at different body mass index (BMI) on pathological complete response (pCR) in breast cancer (BC) patients receiving neoadjuvant chemotherapy (NAC) remains unclear.

**Methods:**

We retrospectively analyzed 834 BC patients who received NAC, categorized into low-BMI (BMI < 25) and high-BMI (BMI ≥ 25) groups. We compared the differences in peripheral blood platelet count (P), lymphocyte count (L), neutrophil count (N), and monocyte count (M) using the Wilcoxon test. Additionally, we calculated the dynamic changes in these immune cells (ΔP, ΔL, ΔN, ΔM) based on hematological tests before and after NAC. Receiver operating characteristic (ROC) curves were used to assess the predictive value of peripheral immune cells for pCR. Logistic regression was used to identify independent risk factors associated with pCR. Finally, a nomogram was constructed based on multivariate logistic regression.

**Results:**

After standardizing peripheral immune cells, before NAC, the P (*P* = 0.008), L (*P* < 0.001), N (*P* < 0.001), and M (*P* = 0.021) were higher in the high-BMI group compared to the low-BMI group. After NAC, the P (*P* = 0.015), L (*P* < 0.001), N (*P* < 0.001), and M (*P* < 0.001) were higher in the high-BMI group compared to the low-BMI group. Compared to before and after NAC, ΔP (0.661) had the highest AUC in predicting pCR for the low-BMI group, while ΔP (0.604), ΔL (0.610), and ΔM (0.607) had the highest AUC in predicting non-pCR for the high-BMI group. ΔP (OR = 3.372, 95% CI: 1.753-6.485, *P* < 0.001) was associated with non-pCR in the low-BMI group; ΔP (OR = 4.435, 95% CI: 1.745-11.267, *P* = 0.002), ΔL (OR = 0.180, 95% CI: 0.052-0.624, *P* = 0.007), and ΔM (OR = 2.267, 95% CI: 1.127-4.558, *P* = 0.022) were independent risk factors associated with non-pCR in the high-BMI group.

**Conclusion:**

Peripheral immune cells in the inflammatory microenvironment at different BMI groups play an important role in predicting pCR in BC patients receiving NAC.

## Introduction

Neoadjuvant chemotherapy (NAC) significantly improves the prognosis in the treatment of primary aggressive breast cancer (BC), primarily reflected in the improvement of disease-free survival and overall survival (1, 2). Pathological complete response (pCR) is the main outcome measure for evaluating the effectiveness of NAC. Body mass index (BMI) is one of the factors that affect the pCR rate (3). However, when obesity is defined by BMI, paradoxical phenomena often occur in clinical outcomes, known as the “obesity paradox” (4). The obesity paradox remains controversial in the context of neoadjuvant treatment for BC. For instance, a meta-analysis reported that a high BMI negatively impacted pCR (5), while other studies showed that high BMI either had no effect or even had a positive effect on pCR (6, 7).

Inflammation is closely linked to cancer, and peripheral blood markers, due to their simplicity, speed, and convenience, are widely used to evaluate clinical outcomes in breast cancer (8). Several studies have reported an association between elevated inflammatory responses and non-pCR (8–10). Complex inflammatory markers, such as neutrophil-lymphocyte ratio (NLR) and platelet-to-lymphocyte ratio (PLR), provide comprehensive inflammatory information (11, 12). However, prior studies have not accounted for the effect of individual immune cells on downstream outcomes (8–12) and have overlooked the true effects of individual immune cells, despite statistical prior hypotheses. Similar to the study by Kuss et al., our study focused on immune cell counts rather than proportions (13). Notably, patients with high BMI often exhibit more pronounced inflammatory responses (14). Therefore, it remains unclear whether the true relationship between BMI and pCR can be explored through peripheral immune cells in the context of the obesity paradox. Moreover, the effects of BMI have not been considered in most inflammatory studies on BC.

To address this gap, we grouped BC patients according to BMI and used peripheral immune cell counts, rather than combinations or ratios of multiple immune cells, to explore the impact of peripheral immune cells on pCR under different BMI groups. Additionally, we calculated the dynamic changes in peripheral immune cells before and after neoadjuvant therapy to further evaluate their predictive value for pCR.

## Materials and methods

### Patients

This study retrospectively analyzed BC patients who underwent surgery after NAC at the Harbin Medical University Cancer Hospital from January 2021 to December 2023. The inclusion criteria were as follows: (1) patients aged 18 years or older; (2) complete NAC Record; (3) patients with complete peripheral hematological examination results before and after NAC; (4) diagnosis of BC confirmed by postoperative histopathology and immunohistochemistry. The exclusion criteria included: (1) distant metastasis; (2) severe infection before surgery; (3) comorbid chronic diseases (such as chronic kidney failure); (4) autoimmune diseases. A total of 834 BC patients were included in the study. All patients had their height and weight measured upon admission. BMI was calculated using the formula: weight (kg)/height (m²). Patients were categorized into low-BMI (BMI < 25) and high-BMI (BMI ≥ 25) groups according to the classification criteria used in previous studies, which are also based on the World Health Organization’s standards.

### Neoadjuvant chemotherapy and pathological evaluation

We used anthracycline-based (A-) and/or taxane-based (T-) neoadjuvant chemotherapy (NAC) regimens, with selected regimens repeated every three weeks. The specific regimens were as follows: AC-T regimen: anthracycline 100mg/m², cyclophosphamide (C) 600mg/m², followed by docetaxel 80-100mg/m²; TAC regimen: taxane 75mg/m², anthracycline 50mg/m², cyclophosphamide 500mg/m²; AT regimen: taxane 75mg/m² and doxorubicin 60mg/m². Other regimens included: AC regimen: anthracycline 90mg/m² and cyclophosphamide 600mg/m²; TC regimen: docetaxel 80-100mg/m² and cyclophosphamide 600mg/m².

Estrogen receptor (ER), progesterone receptor (PR), and HER2 expression status were based on pre-NAC core needle biopsy specimens. Tumors defined as HER2-positive were those with HER2 3+ by immunohistochemistry (IHC) or HER2 2+ with HER2 amplification assessed by fluorescence *in situ* hybridization (FISH). ER and PR were considered positive if more than 1% of invasive tumor cell nuclei showed positive staining. The pCR was defined as the absence of invasive cancer in both the breast and axillary lymph nodes following NAC. All clinical and pathological information of patients was stored in the medical record management system of Harbin Medical University Cancer Hospital.

### Peripheral blood examination

**Figure 1:**
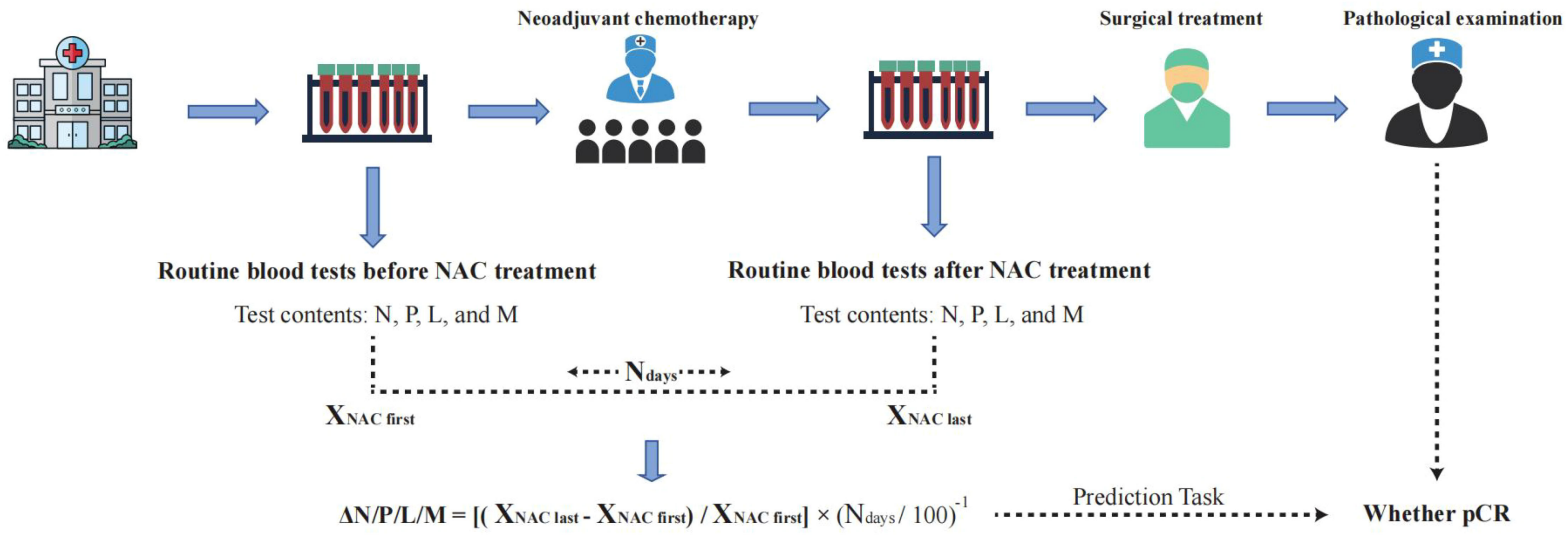
Detailed calculation process of ΔN/P/L/M and prediction task. NAC, neoadjuvant chemotherapy; N, neutrophil count; P, platelet count; L, lymphocyte count; M, monocyte count; pCR, Pathological complete response.

All patients underwent routine hematological tests upon admission. Blood samples were collected from the cubital vein in the morning, after an overnight fast, both before the first NAC and after the last NAC, and sent to the laboratory for analysis. The test results included neutrophil count (N), platelet count (P), lymphocyte count (L), and monocyte count (M). Considering the age heterogeneity of the biological behavior of BC (15), tumors with different invasiveness may be accompanied by varying inflammatory responses. We performed Z-score normalization for N, P, L, and M (16). The formula used was: Z = (x - μ)/σ. Z represents the standardized score adjusted for potential gender and age influences; x is the patient’s N, P, L, or M count; μ is the age-specific mean; and σ is the age-specific standard deviation calculated for the current cohort. Additionally, we calculated the time changes of N, P, L, and M before and after NAC (16). The formula used was: ΔN/P/L/M = [(X_NAC last_ - X_NAC first_)/X_NAC first_] × (N_days_/100) ^- 1^. X_NAC first_ stands for the blood test before NAC treatment, that is, the blood test before any treatment after admission. The test results represent the peripheral inflammatory status of the patient before receiving any treatment. X_NAC last_ represents the preoperative blood routine examination results after the completion of the full course of NAC treatment, and these results reflect the patient’s peripheral inflammatory state after receiving the complete NAC treatment. N_days_ represents the number of days the patient received NAC treatment. Therefore, Δ represents the changes in peripheral blood cell counts before and after NAC treatment, and is used to quantify the alterations in the inflammatory state of patients after receiving NAC therapy. A negative Δ indicates a decrease in blood cell count after NAC treatment, while a positive Δ indicates an increase in blood cell count after NAC treatment. **Figure 1** illustrates the detailed calculation process and prediction task.

### Statistical analysis

The primary outcome of the study was whether pCR occurred. All blood cell counts were presented as median and interquartile range. Categorical variables were expressed as percentages. We used receiver operating characteristic (ROC) curves to calculate the area under the curve (AUC) to evaluate the accuracy of blood cell counts in predicting pCR. Chi-square tests and Wilcoxon tests were used to compare the differences in variables between the low-BMI and high-BMI groups. Additionally, Spearman’s correlation coefficient (r²) was used to assess the correlation between different blood cell counts. Logistic regression was performed to identify independent risk factors associated with pCR, and odds ratios (ORs) and 95% confidence intervals (CIs) were calculated. For logistic regression, we separately analyzed NAC pre-treatment and post-treatment variables including P, L, and NM in the low-BMI and high-BMI groups; as well as P, L, N, and M post-treatment; and ΔP, ΔN, ΔL, ΔM. Furthermore, based on independent risk factors, we used the “rms” package to plot nomograms. Calibration curves and ROC curves were used to evaluate the predictive performance of the nomograms. All analyses were performed using SPSS 25.0 and R 4.3.0. A *P*-value of <0.05 was considered statistically significant.

## Results

### Patient characteristics

A total of 834 BC patients were included in this study, with 215 (25.8%) achieving pCR and 619 (74.2%) not achieving pCR. Patient information is shown in **Table 1**. After grouping based on BMI, there were 493 low-BMI and 341 high-BMI patients. The patients in the low-BMI group (50.33 ± 9.28) were younger than those in the high-BMI group (51.82 ± 8.84) (*P* = 0.021). Regarding pCR, no significant difference in pCR rates was observed between the low-BMI and high-BMI groups (15.7% vs 10.1%, *P* = 0.529).

**Table 1:** Patient characteristics.

Characteristics	Total	Low-BMI	High-BMI	*P* value
n	834	493 (59.1%)	341 (40.9%)	
Age, mean ± sd	50.91 ± 9.12	50.33 ± 9.28	51.82 ± 8.84	**0.021**
Menstrual status, n (%)				0.102
Not postmenopausal	405 (48.6%)	251 (30.1%)	154 (18.5%)	
Menstrual	429 (51.4%)	242 (29%)	187 (22.4%)	
ER, n (%)				0.459
Positive	585 (70.1%)	341 (40.9%)	244 (29.3%)	
Negative	249 (29.9%)	152 (18.2%)	97 (11.6%)	
PR, n (%)				0.951
Positive	493 (59.1%)	291 (34.9%)	202 (24.2%)	
Negative	341 (40.9%)	202 (24.2%)	139 (16.7%)	
HER2, n (%)				0.262
Positive	342 (41%)	210 (25.2%)	132 (15.8%)	
Negative	492 (59%)	283 (33.9%)	209 (25.1%)	
pCR, n (%)				0.529
No	619 (74.2%)	362 (43.4%)	257 (30.8%)	
Yes	215 (25.8%)	131 (15.7%)	84 (10.1%)	
First P, median (IQR)	264 (222, 312)	257 (220, 308)	273 (225, 318)	**0.032**
First L, median (IQR)	1.84 (1.49, 2.26)	1.78 (1.42, 2.19)	1.92 (1.58, 2.39)	**< 0.001**
First N, median (IQR)	3.79 (2.98, 4.85)	3.64 (2.83, 4.71)	4.04 (3.09, 5.10)	**< 0.001**
First M, median (IQR)	0.38 (0.31, 0.47)	0.37 (0.29, 0.46)	0.39 (0.33, 0.48)	**0.028**
Last P, median (IQR)	256 (206, 313)	251 (198, 307)	264 (213, 323)	**0.041**
Last L, median (IQR)	1.35 (1.08, 1.73)	1.3 (1.02, 1.67)	1.43 (1.14, 1.8)	**< 0.001**
Last N, median (IQR)	3.40 (2.50, 4.70)	3.15 (2.34, 4.27)	3.83 (2.85, 5.01)	**< 0.001**
Last M, median (IQR)	0.48 (0.36, 0.61)	0.45 (0.35, 0.58)	0.51 (0.37, 0.65)	**< 0.001**
First P Z-score, median (IQR)	-0.09 (-0.70, 0.58)	-0.16 (-0.78, 0.50)	0.07 (-0.60, 0.63)	**0.008**
First L Z-score, median (IQR)	-0.11 (-0.74, 0.64)	-0.19 (-0.82, 0.53)	0.03 (-0.57, 0.89)	**< 0.001**
First N Z-score, median (IQR)	-0.14 (-0.66, 0.53)	-0.26 (-0.74, 0.36)	-0.01 (-0.56, 0.70)	**< 0.001**
First M Z-score, median (IQR)	-0.11(-0.67, 0.51)	-0.13 (-0.74, 0.51)	-0.04 (-0.53, 0.63)	**0.021**
Last P Z-score, median (IQR)	-0.08 (-0.70, 0.58)	-0.16 (-0.78, 0.51)	0.04(-0.60, 0.69)	**0.015**
Last L Z-score, median (IQR)	-0.17 (-0.67, 0.51)	-0.24 (-0.81, 0.45)	-0.02(-0.58, 0.65)	**< 0.001**
Last N Z-score, median (IQR)	-0.21 (-0.59, 0.35)	-0.31 (-0.68, 0.20)	-0.06(-0.46, 0.46)	**< 0.001**
Last M Z-score, median (IQR)	-0.14 (-0.67, 0.46)	-0.26 (-0.71, 0.33)	-0.01 (-0.59, 0.55)	**< 0.001**
ΔP, median (IQR)	0.28 (0.06, 0.47)	0.28(0.06, 0.46)	0.28 (0.07, 0.48)	0.515
ΔL, median (IQR)	-0.17 (-0.27, -0.05)	-0.15 (-0.26, -0.05)	-0.18 (-0.28, -0.05)	0.195
ΔN, median (IQR)	-0.07 (-0.23, 0.13)	-0.08(-0.23, 0.12)	-0.06(-0.23, 0.13)	0.518
ΔM, median (IQR)	0.14 (-0.03, 0.44)	0.14 (-0.05, 0.43)	0.15 (0, 0.47)	0.174

pCR: Pathological complete response. BMI: Body mass index. ER: Estrogen receptor. PR: Progesterone receptor. P: Platelet count. L: Lymphocyte count. N: Neutrophil count. M: Monocyte count. Z-score: Age-specific adjustments. Δ: Dynamic Changes.

Values with p < 0.05 are bolded.

### Peripheral blood cell count difference

Significant differences in peripheral blood cell counts were observed between the low-BMI and high-BMI groups (**Table 1**). For the original peripheral hematological tests, before NAC, the high-BMI group had higher P (273 vs 257, *P* = 0.032), L (1.92 vs 1.78, *P* < 0.001), N (4.04 vs 3.64, *P* < 0.001), and M (0.39 vs 0.37, P = 0.028) compared to the low-BMI group. After NAC, the high-BMI group had higher P (264 vs 251, *P* = 0.041), L (1.43 vs 1.30, *P* < 0.001), N (3.83 vs 3.15, *P* < 0.001), and M (0.51 vs 0.45, *P* = 0.028) compared to the low-BMI group.

**Figure 2:**
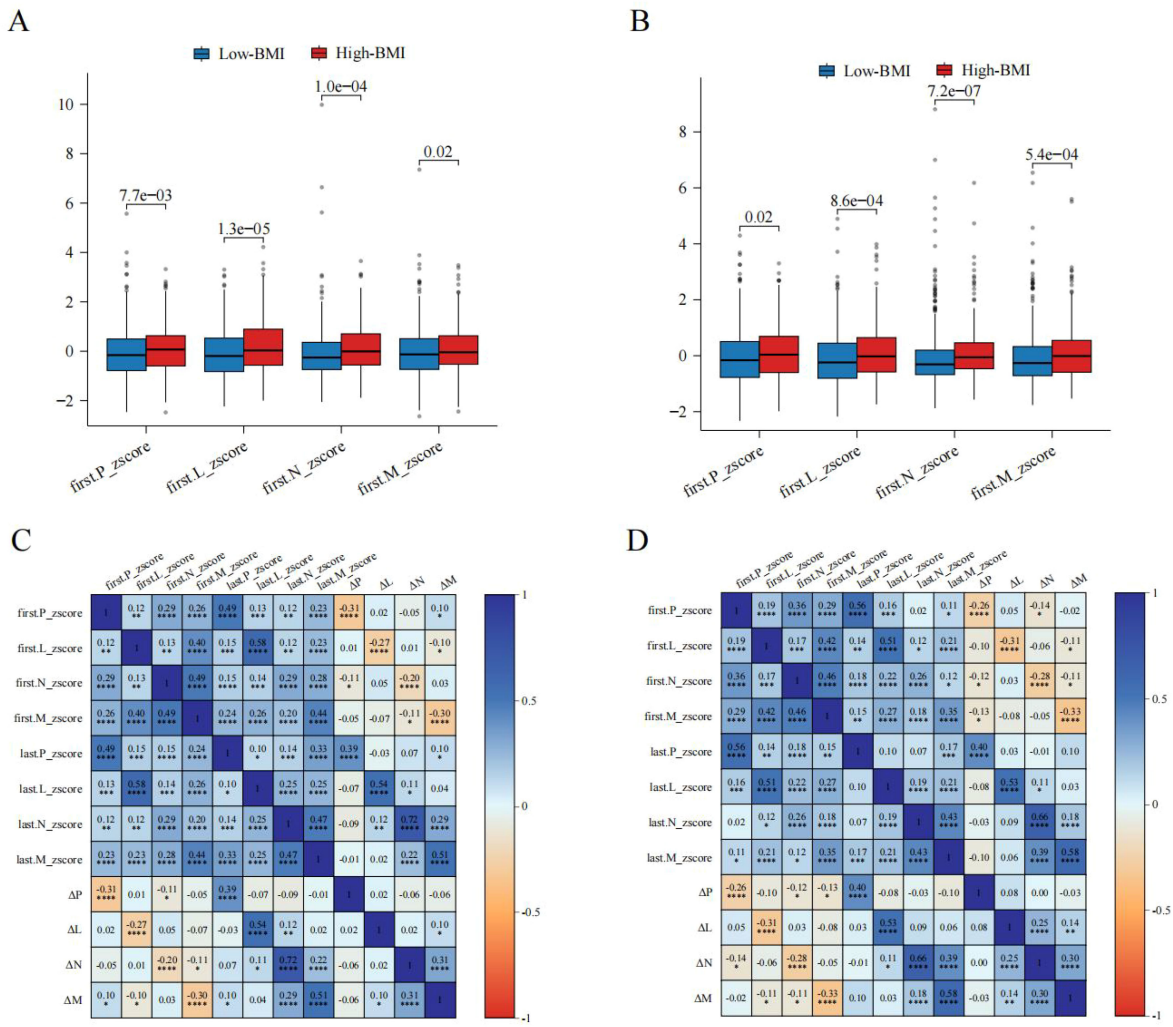
Differences and correlations of peripheral blood cells in different BMI groups. **(A)** Differences in peripheral immune cells in low-BMI group. **(B)** Differences in peripheral immune cells in high-BMI group. **(C)** Correlations of peripheral blood cells in low-BMI group. **(D)** Correlations of peripheral blood cells in high-BMI group. BMI, Body mass index; P, Platelet count; L, Lymphocyte count; N, Neutrophil count; M, Monocyte count; Z-score, Age-specific adjustments; Δ, Dynamic Changes. *: p value<0.05; **: p value < 0.01; ***: p value <0.005; ****: p value <0.001

After standardization, for the pre-NAC hematological examination, the high-BMI group had higher P Z-score (0.07 vs -0.16, *P* = 0.008), L Z-score (0.03 vs -0.19, *P* < 0.001), N Z-score (-0.01 vs -0.26, *P* < 0.001), and M Z-score (-0.04 vs -0.13, *P* = 0.021) compared to the low-BMI group (**Figure 2A**). After NAC, the high-BMI group had higher P Z-score (0.04 vs -0.16, P = 0.015), L Z-score (0.02 vs -0.24, *P* < 0.001), N Z-score (-0.06 vs -0.31, *P* < 0.001), and M Z-score (-0.01 vs -0.26, *P* < 0.001) compared to the low-BMI group (**Figure 2B**). However, no significant differences were observed in the changes in blood cell counts before and after NAC between the low-BMI and high-BMI groups (**Table 1**).

Additionally, for the low-BMI group, significant correlations were observed for P, N, L, and M before and after NAC, with significant negative correlations between pre-NAC P, N, L, and M and ΔP, ΔN, ΔL, and ΔM (**Figure 2C**). For the high-BMI group, significant correlations were also observed for P, N, L, and M before and after NAC, with significant negative correlations between pre-NAC P, N, L, and M and ΔP, ΔN, ΔL, and ΔM (**Figure 2D**).

### Evaluation of the diagnostic performance of blood cell count

**Figure 3:**
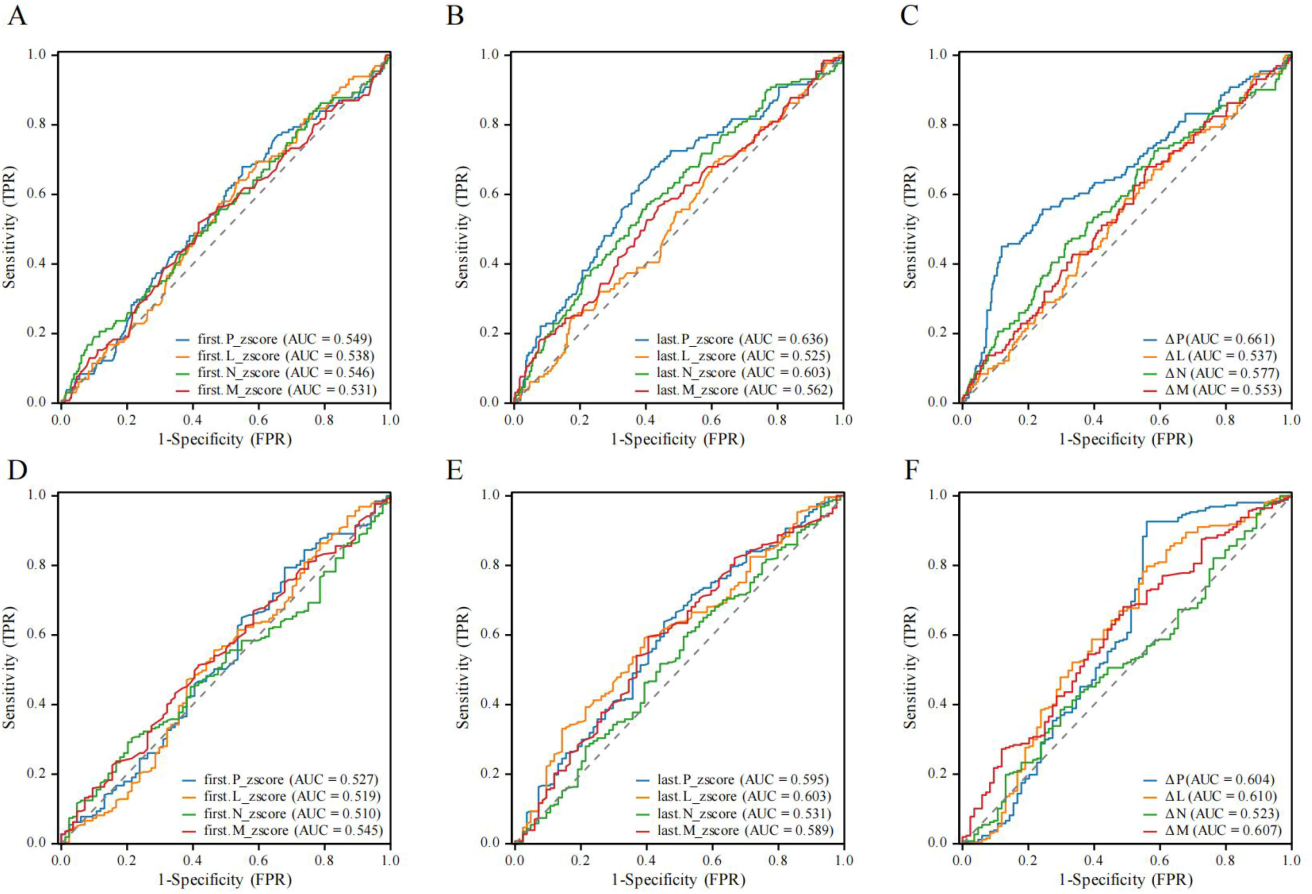
ROC curve for predicting non-pCR. **(A)** ROC curve for predicting non-pCR before NAC in the low-BMI group. **(B)** ROC curve for predicting non-pCR after NAC in the low-BMI group. **(C)** ROC curve of dynamic changes of peripheral immune cells predicting non-pCR in the low-BMI group. **(D)** ROC curve for predicting non-pCR before NAC in the high-BMI group. **(E)** ROC curve for predicting non-pCR before NAC in the high-BMI group. **(F)** ROC curve of dynamic changes of peripheral immune cells predicting non-pCR in the high-BMI group. ROC, Receiver operating characteristic curve; pCR, Pathological complete response; BMI, Body mass index; P, Platelet count; L, Lymphocyte count; N, Neutrophil count; M, Monocyte count; Z-score, Age-specific adjustments; Δ, Dynamic Changes.

In the low-BMI group, compared to N, L, and M, the P before and after NAC had the highest AUC (0.549 and 0.636) (**Figures 3A, B**), and in dynamic changes, ΔP had the highest AUC (0.661) (**Figure 3C**). This indicates that the dynamic changes in P are superior to single P in predicting pCR in the low-BMI group. In the high-BMI group, the AUC of P (0.595 vs 0.592), L (0.603 vs 0.519), N (0.531 vs 0.510), and M (0.589 vs 0.549) after NAC was higher than before NAC (**Figures 3D, E**). The AUC of ΔP (0.604), ΔL (0.610), and ΔM (0.607) was higher than that of pre- or post-NAC, with ΔP (0.610) having the highest AUC (**Figure 3F**). This suggests that the dynamic changes in P, L, and M are superior to single P, L, and M in predicting pCR in the high-BMI group.

### Univariate and multivariate logistic analysis for predicting pCR

For the low-BMI group, pre-NAC P, L, N, and M were not associated with pCR. Post-NAC P was significantly associated with non-pCR (OR = 1.56, 95% CI: 1.250-1.950, *P* < 0.001) (**Table 2**). ΔP (OR = 3.847, 95% CI: 2.070-7.150, *P* < 0.001) was significantly associated with non-pCR (**Table 2**). For the high-BMI group, pre-NAC P, L, N, and M were not associated with pCR. Post-NAC P (OR = 1.357, 95% CI: 1.022-1.801, *P* = 0.035), L (OR = 0.606, 95% CI: 0.469-0.785), and M (OR = 1.478, 95% CI: 1.089-2.006, *P* = 0.012) were significantly associated with non-pCR (**Table 2**). ΔP (OR = 4.385, 95% CI: 1.833-10.489, *P* < 0.001), ΔL (OR = 0.127, 95% CI: 0.038-0.431, *P* < 0.001), and ΔM (OR = 3.011, 95% CI: 1.511-5.998, *P* = 0.002) were significantly associated with non-pCR (**Table 2**).

**Table 2:** Univariate and multivariate logistic regression analysis of peripheral immune cells in predicting non-pCR.

Characteristics	Univariate analysis	*P* value	Multivariate analysis	*P* value
	OR (95% CI)		OR (95% CI)	
Low-BMI				
first.P_zscore	1.108 (0.906 – 1.355)	0.318		
first.L_zscore	1.154 (0.931 – 1.431)	0.192		
first.N_zscore	1.085 (0.886 – 1.329)	0.430		
first.M_zscore	1.082 (0.886 – 1.321)	0.441		
last.P_zscore	1.561 (1.250 – 1.950)	**< 0.001**	1.561 (1.250 – 1.950)	**< 0.001**
last.L_zscore	0.929 (0.760 – 1.136)	0.474		
last.N_zscore	1.195 (0.956 – 1.494)	0.118		
last.M_zscore	1.250 (0.996 – 1.569)	0.055		
ΔP	3.847 (2.070 – 7.150)	**< 0.001**	3.847 (2.070 – 7.150)	**< 0.001**
ΔL	0.512 (0.183 – 1.431)	0.202		
ΔN	1.196 (0.816 – 1.753)	0.359		
ΔM	1.176 (0.824 – 1.678)	0.372		
High-BMI				
first.P_zscore	1.136 (0.880 – 1.468)	0.328		
first.L_zscore	0.976 (0.768 – 1.241)	0.843		
first.N_zscore	0.935 (0.708 – 1.235)	0.637		
first.M_zscore	1.166 (0.902 – 1.508)	0.24		
last.P_zscore	1.373 (1.046 – 1.803)	**0.023**	1.357 (1.022 – 1.801)	**0.035**
last.L_zscore	0.686 (0.541 – 0.871)	**0.002**	0.606 (0.469 – 0.785)	**< 0.001**
last.N_zscore	1.020 (0.770 – 1.351)	0.892		
last.M_zscore	1.379 (1.032 – 1.844)	**0.03**	1.478 (1.089 – 2.006)	**0.012**
ΔP	3.389 (1.478 – 7.773)	**0.004**	4.385 (1.833 – 10.489)	**< 0.001**
ΔL	0.225 (0.067 – 0.758)	**0.016**	0.127 (0.038 – 0.431)	**< 0.001**
ΔN	0.692 (0.440 – 1.088)	**0.111**		
ΔM	2.127 (1.125 – 4.024)	**0.020**	3.011 (1.511 – 5.998)	**0.002**

pCR: Pathological complete response. BMI: Body mass index. P: Platelet count. L: Lymphocyte count. N: Neutrophil count. M: Monocyte count. Z-score: Age-specific adjustments. Δ: Dynamic Changes.

Values with p < 0.05 are bolded.

**Figure 4:**
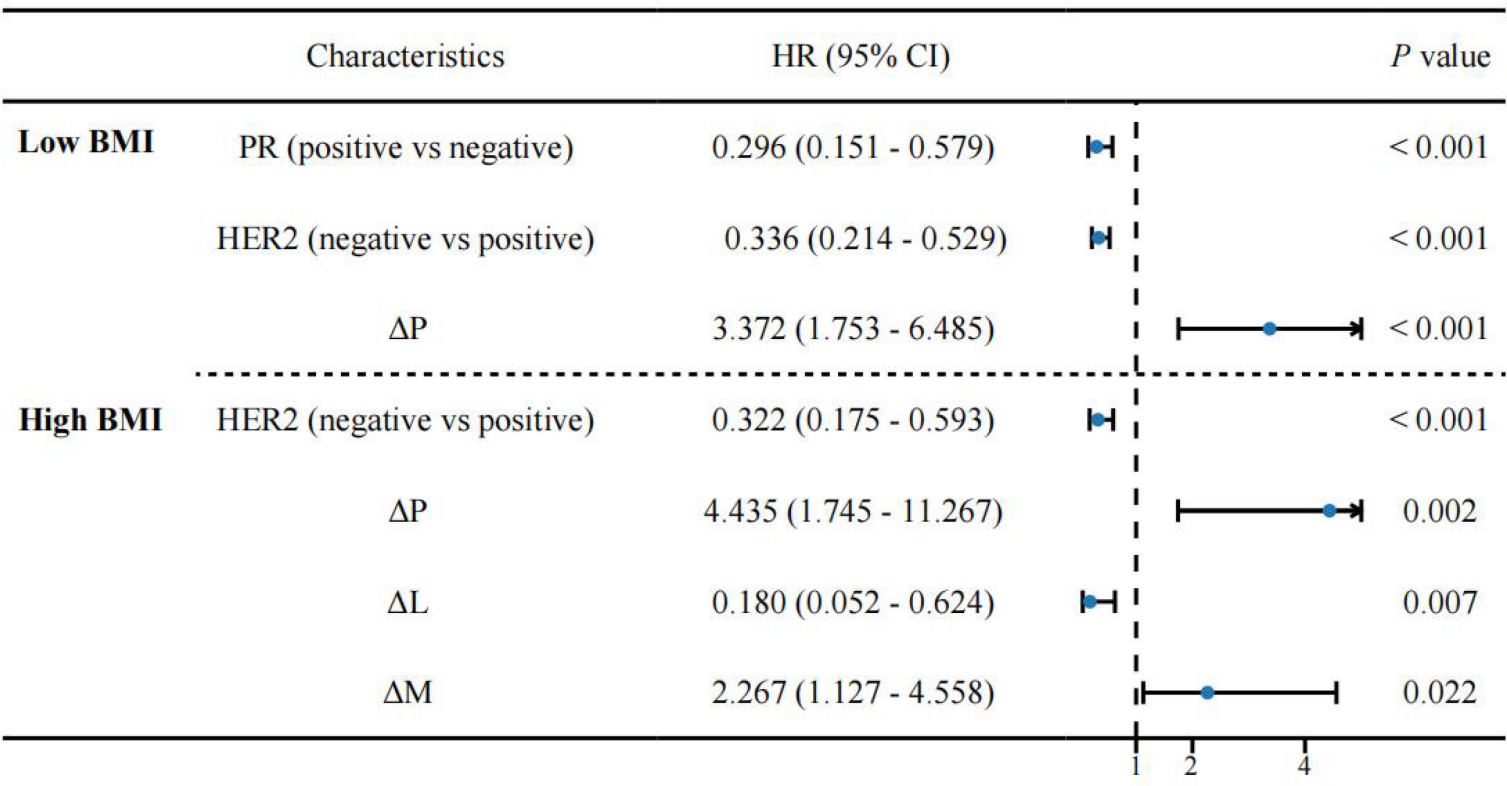
Multivariate regression analysis for predicting non-pCR. P, Platelet count; L, Lymphocyte count; M, Monocyte count; Δ, Dynamic Changes.

Therefore, considering the significant role of dynamic changes in peripheral blood cells for predicting pCR, we performed logistic regression based on these dynamic changes. **Table 3** presents the independent risk factors associated with pCR in different BMI groups. Subsequently, we incorporated the significant factors (*P* < 0.05) into the multivariate analysis. For the low-BMI group, PR (OR = 0.296, 95% CI: 0.151-0.579, *P* < 0.001), HER2 (OR = 0.336, 95% CI: 0.214-0.529, *P* < 0.001), and ΔP (OR = 3.372, 95% CI: 1.753-6.485, *P* < 0.001) were independent risk factors associated with non-pCR (**Figure 4**). For the high-BMI group, HER2 (OR = 0.322, 95% CI: 0.175-0.593, *P* < 0.001), ΔP (OR = 4.435, 95% CI: 1.745-11.267, *P* = 0.002), ΔL (OR = 0.180, 95% CI: 0.052-0.624, *P* = 0.007), and ΔM (OR = 2.267, 95% CI: 1.127-4.558, *P* = 0.022) were independent risk factors associated with non-pCR (**Figure 4**).

**Table 3:** Univariate regression analysis for predicting non-pCR.

Characteristics	Low BMI		High BMI	
	Odds ratio (95% CI)	P value	Odds ratio (95% CI)	P value
Age	Odds Ratio (95% CI)	P value	0.989 (0.962 – 1.017)	0.442
ER (positive vs negative)	1.004 (0.982 – 1.026)	0.737	0.336 (0.200 – 0.565)	**< 0.001**
PR (positive vs negative)	0.352 (0.232 – 0.534)	**< 0.001**	0.385 (0.233 – 0.637)	**< 0.001**
HER2 (negative vs positive)	0.247 (0.162 – 0.377)	**< 0.001**	0.210 (0.124 – 0.356)	**< 0.001**
Menstrual status (not postmenopausal vs menopause)	0.248 (0.162-0.380)	**< 0.001**	0.557 (0.334 – 0.929)	**0.025**
ΔP	0.971 (0.651-1.449)	0.887	3.389 (1.478 – 7.773)	**0.004**
ΔL	3.847 (2.070 – 7.150)	**< 0.001**	0.225 (0.067 – 0.758)	**0.016**
ΔN	0.512 (0.183 – 1.431)	0.202	0.692 (0.440 – 1.088)	0.111
ΔM	1.196 (0.816 – 1.753)	0.359	2.127 (1.125 – 4.024)	**0.020**

pCR: Pathological complete response. BMI: Body mass index. ER: Estrogen receptor. PR: Progesterone receptor. P: Platelet count. L: Lymphocyte count. N: Neutrophil count. M: Monocyte count. Z-score: Age-specific adjustments. Δ: Dynamic Changes.

Values with p < 0.05 are bolded.

### Nomogram for predicting pCR

**Figure 5:**
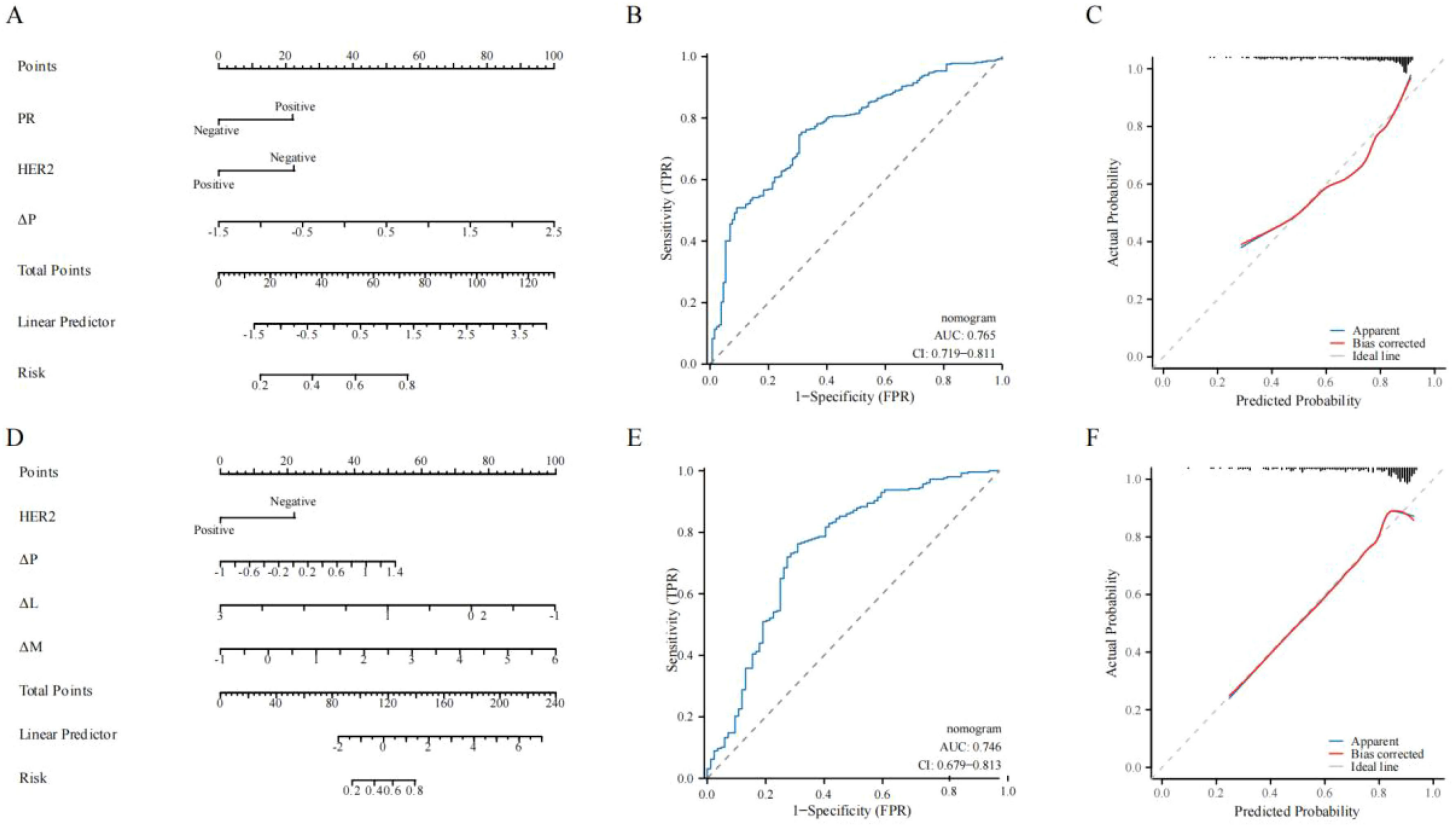
Nomogram for predicting non-pCR in low-BMI and high-BMI. **(A, D)** Nomogram construction for low-BMI and high-BMI. **(B, E)** ROC curves of the nomograms for low-BMI and high-BMI. **(C, F)** Calibration plot of the nomogram for low-BMI and high-BMI. ROC: Receiver operating characteristic curve. BMI, Body mass index; PR, Progesterone receptor; P, Platelet count; L, Lymphocyte count; M, Monocyte count; Δ, Dynamic Changes.

Based on the multivariate logistic results for low-BMI and high-BMI, we separately plotted nomograms to predict non-pCR for the low-BMI and high-BMI groups (**Figures 5A, D**). For low-BMI, the nomogram had an AUC of 0.765 in predicting non-pCR (**Figure 5B**), and the calibration curve showed good agreement between the predicted and actual probabilities (**Figure 5C**). For high-BMI, the nomogram had an AUC of 0.746 in predicting non-pCR (**Figure 5E**), and the calibration curve also demonstrated good agreement between the predicted and actual probabilities (**Figure 5F**).

## Discussion

In this study, we explored the effects of P, L, N, and M and dynamic changes on pCR in the low-BMI and high-BMI group before and after NAC. The results showed that elevated P was correlated with non-pCR in the low-BMI group. The increased P and M and the reduced L are correlated with non-pCR in the high-BMI group. In addition, we calculated the dynamic changes of P, L, N, and M based on hematological examinations before and after NAC, and the results showed that the increase of ΔP was correlated with non-pCR in the low-BMI group; the increase of ΔP and ΔM, and the decrease of ΔL were correlated with non-pCR in the high-BMI group. Our results show that BMI is not significantly associated with pCR, whereas pCR with different BMIs may be affected by the immune status of the patient’s peripheral blood, and peripheral blood cells may dominate the obesity paradox of pCR.

Regarding the relationship between BMI and pCR, we found that BMI was not associated with pCR. Similar to the study by Warner et al., obesity did not affect pCR in BC (OR = 0.86, 95% CI: 0.64-1.16) (7). In contrast, a report by Farr et al. suggested that obesity contributed to an increased pCR rate (OR = 4.29, 95% CI: 1.42-13.91) (6), which contradicts the results of a meta-analysis (5). A study from East China also reported that obesity had no effect on pCR (17). These findings seem to suggest that, in Asian populations, BMI may not significantly influence pCR, while the impact of BMI on pCR remains contradictory in European and American populations. Additionally, pCR rates vary across different ethnic groups (18), which indirectly implies that the pCR rate in BC may differ due to ethnicity-specific nutrition. Moreover, age may be related to increased BMI (19), and BC also exhibits heterogeneous invasiveness across different age groups (15), further complicating the relationship between BMI and pCR. Unfortunately, previous studies did not adjust for peripheral blood tests (11, 12, 20, 21) or group BMI.

It is known that lipid metabolism participates in the occurrence of various tumors and disease progression by altering lipid synthesis, storage, and catabolism (22, 23). Lipid alterations may arise as a consequence of cancer treatment and may have a role in treatment resistance. For example, in the lipid metabolism and homeostasis of patients with neuroendocrine tumors: lipid metabolism has been explored as a factor ranging from risk factors to potential therapeutic targets (23). This implies that there is still considerable room for exploration in the relationship between lipid metabolism and cancer prognosis. Adipose tissue secretes various adipokines that promote lymphocyte infiltration (24). These findings are consistent with the results we observed. We found that patients in the high-BMI group had higher levels of inflammation. This suggests that low-BMI and high-BMI groups are associated with different inflammatory microenvironments. Our multivariate results indicate that P is a risk factor for pCR in the low-BMI group, while P, L, and M are risk factors for pCR in the high-BMI group. This further highlights the importance of the inflammatory microenvironment in the high-BMI group. A consistent finding is that increased P is associated with non-pCR, which is in line with the results of Lusho et al. (25). P promotes cancer progression and metastasis through the secretion of platelet-derived growth factors (PDGF) and vascular endothelial growth factor (VEGF) (26, 27). Cancer cells induce megakaryocyte differentiation into platelets via inflammatory mediators (28), which further exacerbates the mutual stimulation between P and tumor cells. Additionally, we observed significant correlations between P and N, L, and M, which further supports the existence of a complex regulatory network within the inflammatory microenvironment.

In the high-BMI group, elevated P, L, and M were associated with non-pCR, while N was not associated with pCR. This may be related to the lower incidence of neutropenia in obese women (29). L plays an important role in immune surveillance and the suppression of tumor cell proliferation (30), and increased L is associated with better BC outcomes (31). M promotes tumor growth by differentiating into tolerogenic dendritic cells that produce interleukin-10 (32), and circulating M are recruited to primary tumors and metastatic sites, where they differentiate into M2 phenotype tumor-associated macrophages under the influence of chemokines, leading to immune suppression (33). These mechanisms may explain the impact of peripheral immunity on pCR.

Unlike previous studies, we assessed the dynamic changes in P, L, N, and M and their impact on pCR. Dan et al. evaluated pCR rates using the dynamic changes in NLR (34). Dynamic changes help clinicians to continuously and individually monitor the patient’s condition in real-time. Although using ratios of multiple immune cells provides more inflammatory information, it may overlook the true impact of individual immune cells. Therefore, we used ΔP, ΔL, ΔN, and ΔM to predict pCR. The results showed that elevated ΔP was associated with non-pCR in the low-BMI group; elevated ΔP and ΔM, and decreased ΔL were associated with non-pCR in the high-BMI group. This important finding further reinforces our results regarding individual immune cells before and after NAC. Increased inflammatory response or immune suppression during treatment is unfavorable for pCR. Furthermore, we constructed a nomogram to predict pCR based on dynamic changes. The nomogram predicted AUCs of 0.765 and 0.746 for low-BMI and high-BMI, respectively, with good agreement between the predicted and actual probabilities. These results further emphasize the predictive value of single peripheral immune cells in predicting pCR and highlight the prominent role of peripheral immune cells in the obesity paradox.

However, there are some limitations to this study. First, due to the retrospective nature of this study, the conclusions need to be validated in prospective studies. Second, this study focused only on the BC population in Northeast China, and the applicability of the results to different ethnic groups remains to be determined.

## Conclusion

In conclusion, we explored the impact of the inflammatory microenvironment in low-BMI and high-BMI groups on pCR in BC patients receiving NAC. Elevated ΔP was associated with non-pCR in the low-BMI group, while increased ΔP and ΔM, along with decreased ΔL, were associated with non-pCR in the high-BMI group. These results further reveal that immune cells may play a dominant role in the obesity paradox.

## Data Availability

The raw data supporting the conclusions of this article will be made available by the authors, without undue reservation.
